# CNS neuroblastoma, *FOXR2*-activated and its mimics: a relevant panel approach for work-up and accurate diagnosis of this rare neoplasm

**DOI:** 10.1186/s40478-023-01536-7

**Published:** 2023-03-14

**Authors:** Arnault Tauziède-Espariat, Dominique Figarella-Branger, Alice Métais, Emmanuelle Uro-Coste, Claude-Alain Maurage, Benoît Lhermitte, Aude Aline-Fardin, Lauren Hasty, Alexandre Vasiljevic, Dan Chiforeanu, Guillaume Chotard, Homa Adle-Biassette, Alexandra Meurgey, Raphaël Saffroy, Delphine Guillemot, Gaëlle Pierron, Philipp Sievers, Pascale Varlet, the RENOCLIP-LOC

**Affiliations:** 1 Department of Neuropathology, GHU Paris-Psychiatrie et Neurosciences, Sainte-Anne Hospital, 1, rue Cabanis, 75014 Paris, France; 2 INSERM, UMR 1266, IMA-Brain, Institut de Psychiatrie et Neurosciences de Paris, Paris, France; 3 Department of Anatomopathology and Neuropathology, CNRS, INP, Inst Neurophysiopathol, and APHM, La Timone Hospital, Aix-Marseille Univ, 13385 Marseille, France; 4 Department of Pathology, Toulouse University Hospital, Toulouse, France; 5 Cancer Research Center of Toulouse (CRCT), INSERM U1037, Toulouse, France; 6 Université Paul Sabatier, Toulouse III, Toulouse, France; 7 Institute of Pathology, Centre de Biologie Pathologie, Lille University Hospital, 59000 Lille, France; 8 Department of Pathology, Strasbourg Hospital, Strasbourg, France; 9 Department of Pathology, Fort-de-France Hospital, Fort-de-France, France; 10 Department of Pathology and Neuropathology, GHE, Hospices Civils de Lyon, Lyon, France; 11 Department of Pathology, Ponchaillou Hospital, Rennes, France; 12 Department of Pathology, Pellegrin Hospital, Bordeaux, France; 13 Department of Pathology, Lariboisière Hospital, APHP, 75475 Paris, France; 14 Department of Biopathology, Léon Bérard Cancer Center, Lyon, France; 15 Department of Biochemistry and Oncogenetics, Paul Brousse Hospital, 94804 Villejuif, France; 16 Curie Institute Research Center, INSERMU830, Paris-Sciences-Lettres, Paris, France; 17 Laboratory of Somatic Genetics, Curie Institute Hospital, Paris, France; 18 Department of Neuropathology, Institute of Pathology, University Hospital Heidelberg, Heidelberg, Germany; 19 Clinical Cooperation Unit Neuropathology, German Consortium for Translational Cancer Research (DKTK), German Cancer Research Center DKFZ, Heidelberg, Germany

The “neuroblastoma of the central nervous system” (CNS) has been referenced in the World Health Organization’s (WHO) classification of brain tumors since 1993 [2]. However, in recent years, thanks to molecular analyses, *forkhead box R2 *(*FOXR2*) alterations have become associated with this tumor type and as a result, the WHO classification renamed the neoplasm “CNS neuroblastoma (NB), *FOXR2-*activated” [4, 5]. These alterations are comprised of complex structural rearrangements for which routine testing is not easily implemented [4]. Consequently, the diagnosis is confirmed using advanced molecular techniques (Next-generation sequencing or DNA-methylation profiling) [3]. In this context, routine biomarkers for neuropathologists are needed to facilitate a NB-FOXR2 diagnosis. The aims of this report are to present the French national RENOCLIP-LOC network’s experience diagnosing NB-FOXR2 and to propose an alternative diagnostic approach.

Our study included a total of 24 cases initially suspected to be NB-FOXR2, based on histopathology alone, and reviewed by the French national network. All tumors were pediatric (except one in a young adult) and located in the supratentorial region. We performed an immunohistochemical (IHC) panel (including Olig2, synaptophysin, vimentin, and SOX10), fluorescent in situ (FISH) analysis of chromosome 1, and DNA-methylation profiling for all tumors. Each maintained BRG1 and INI1 expression and showed no immunoexpression for Lin28A.

DNA-methylation profiling using the CNS tumor Classifier supported the NB-FOXR2 diagnosis in 9/24 cases (37%), with a calibrated score (> 0.9) for nine of the samples. The other diagnoses having scores > 0.9 included: high-grade gliomas, *IDH-* and H3-wildtype (n = 2); glioneuronal tumors not otherwise specified, subtype A (n = 2); a diffuse glioneuronal tumor with oligodendroglioma-like features and nuclear clusters (DGONC); an anaplastic neuroepithelial tumor with condensed nuclei (ANTCON) [1]; a CNS embryonal tumor with *BRD4::LEUTX* fusion; a CNS tumor *BCOR*-altered; and a neuroepithelial tumor, *PLAGL1*-fused. An orthogonal validation, using different molecular techniques, confirmed these diagnoses (cf. Additional file 1: Table 1). The six remaining cases presented a low calibrated score for different methylation classes. All cases were included in a t-distributed stochastic neighbor embedding analysis to better characterize tumors with low scores. Finally, 10/24 cases were confirmed as NB-FOXR2.

From these results, we tested the sensitivity/specificity of different technical approaches using IHC, FISH, or combinations of these different techniques. The best diagnostic panel included an IHC panel (showing Olig2, synaptophysin, and SOX10 immunopositivities without vimentin immunoexpression), and FISH analysis (presence of 1q gain) with a very high sensitivity/specificity (Fig. 1 and Additional file 2: Figure 1).

**Fig. 1:**
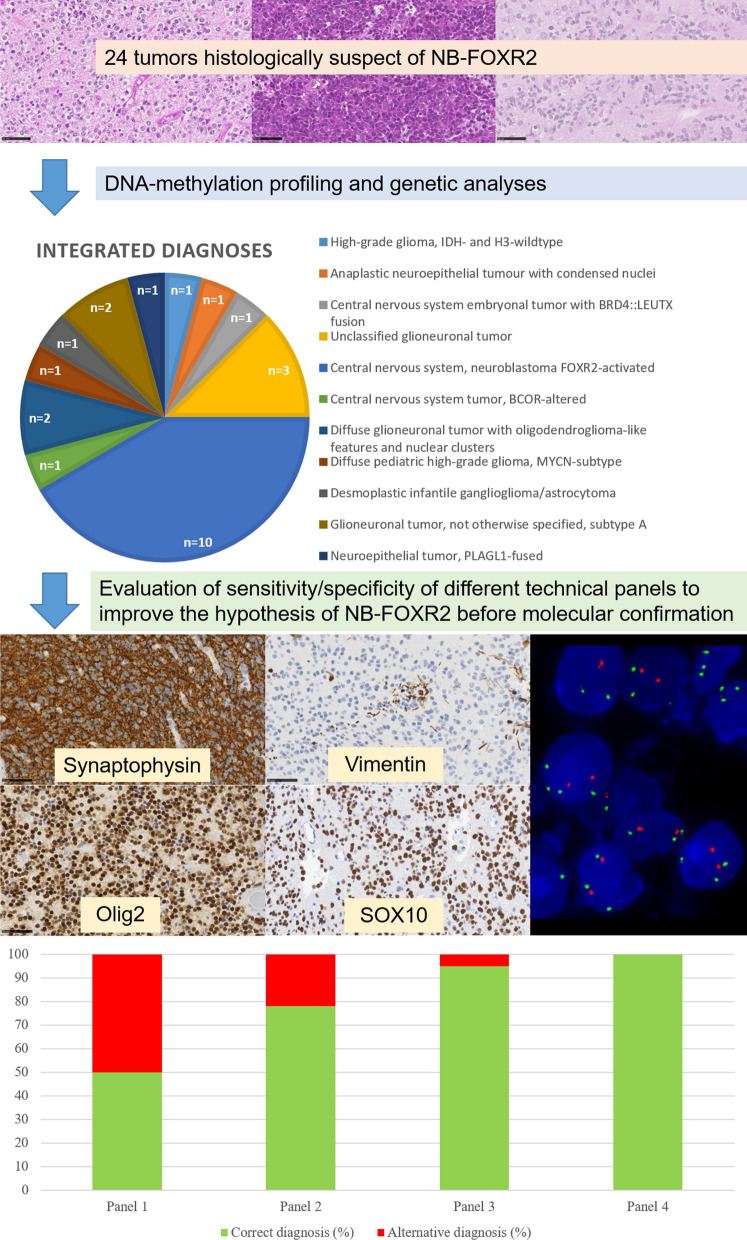
Flowchart of the study. Different technical panels used: panel 1 (Olig2 and synaptophysin immunopositivities, and vimentin negativity); panel 2 (Olig2, synaptophysin and SOX10 immunopositivities); panel 3 (Olig2, synaptophysin and SOX10 immunopositivities, and vimentin negativity); panel 4 (Olig2, synaptophysin and SOX10 immunopositivities, and vimentin negativity, and 1q gain). The sensitivity and specificity for each panel were: 100 and 71% (panel 1); 100 and 50% (panel 2); 100 and 86% (panel 3); and 100 and 100% (panel 4)

In accordance with the WHO Classification, our work evidenced that a diagnosis of NB-FOXR2 based on morphology alone is currently not possible. Indeed, many differential heterogeneous diagnostic pitfalls exist, including glial, glioneuronal, embryonal and new emerging tumor types. Many of these have been very recently described in the literature and are not yet listed in the latest WHO classification, highlighting the velocity with which CNS tumors are being deciphered in recent years thanks to DNA-methylation profiling, and thus, their increasing complexity. The essential diagnostic criteria of the WHO classification defines NB-FOXR2 as an embryonal tumor having foci of neuroblastic or neuronal differentiation and a *FOXR2* activation or a DNA-methylation profile aligned with this diagnosis. These genetic or epigenetic diagnostic techniques are not routinely used or available in all countries worldwide. However, IHC and FISH analyses are well established, can be automated, are relatively easy to standardize, are less expensive, and are widely available in most pathology laboratories worldwide. In this context, the national French histopathological network’s experience showed that IHC (confirming the necessity of a co-expression of Olig2 and synaptophysin, and the interest of SOX10 immunopositivity) and FISH analyses (confirming the necessity of chromosome 1q gain) may improve the diagnosis of NB-FOXR2, and help eliminate potential mimickers [3, 4].

In conclusion, the diagnosis of NB-FOXR2 may be ameliorated by using an algorithmic approach that includes several criteria based on histopathology, IHC and FISH analysis. This diagnostic panel can be tested in further series in order to be validated as a working formula that facilitates the diagnostic approach and reaches an accurate diagnosis in a resource-limited environment.

## Supplementary Information

**Additional file 1.** Histopathological and molecular features of differential diagnoses of CNS neuroblastoma, *FOXR2*-activated.

**Additional file 2: Figure 1.** Immunohistochemical and FISH analyses results of the differential diagnoses.
