# Supplementary figures and images for: CNS neuroblastoma, FOXR2-activated and its mimics: a relevant panel approach for work-up and accurate diagnosis of this rare neoplasm
Source: Acta Neuropathol Commun. 2023 Mar 14;11:43. doi: 10.1186/s40478-023-01536-7 (PMC10012567; doi:10.1186/s40478-023-01536-7)

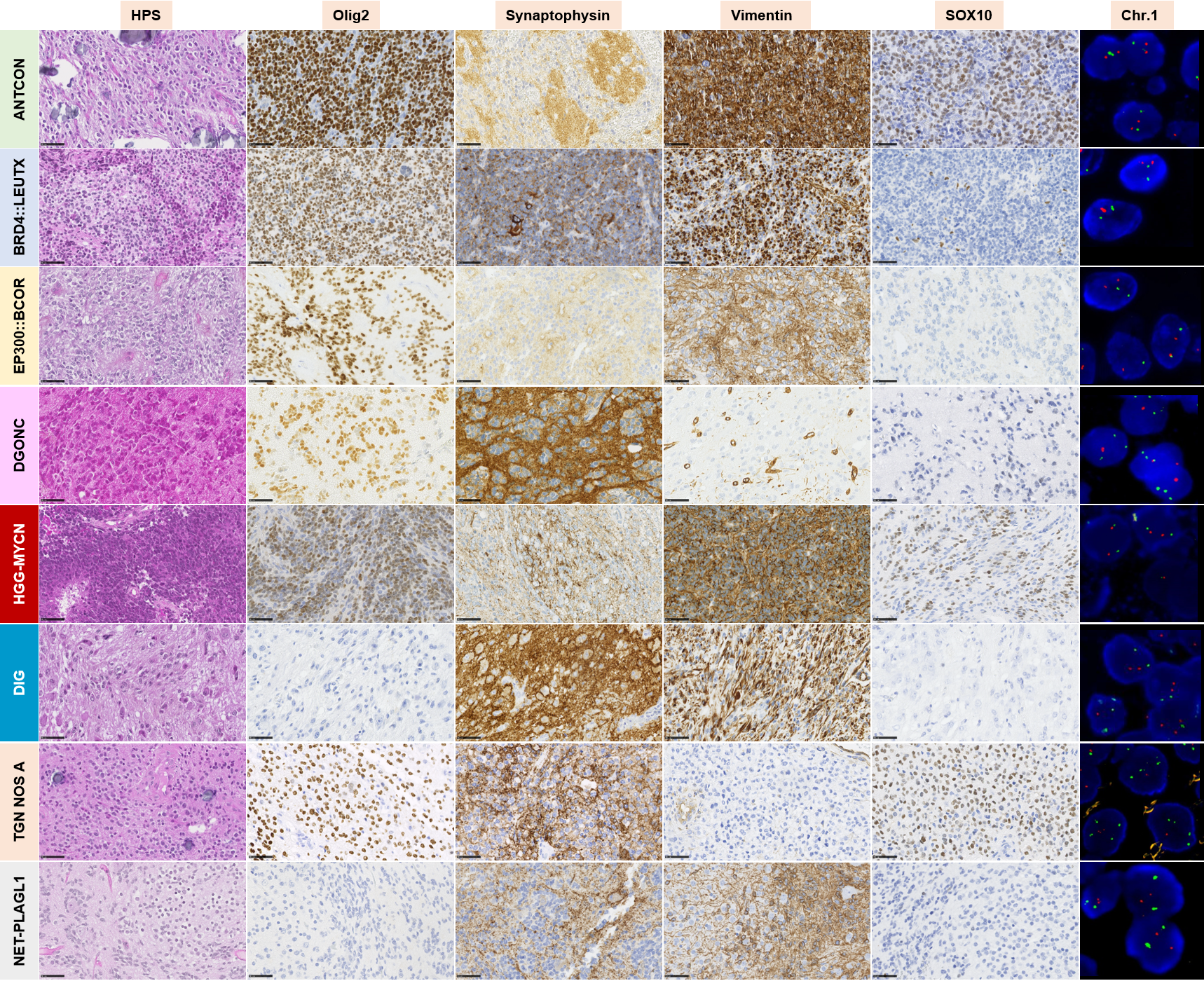

Supplement: Supplementary file 2 — Additional file 2: Figure 1. Immunohistochemical and FISH analyses results of the differential diagnoses. [file 40478_2023_1536_MOESM2_ESM.tif]
